# Dystrophin and dystrophin association protein expression decreases with age in vascular smooth muscle

**DOI:** 10.1007/s11357-025-01947-5

**Published:** 2025-11-01

**Authors:** Katherine M. Kaplan, Yueyue Xiong, Audrey Lucerne, Kathleen G. Morgan

**Affiliations:** 1 Department of Molecular Biology, Cell Biology, and Biochemistry, Boston University, Boston, MA USA; 2 Department of Physiology, Boston University, Boston, MA USA

**Keywords:** Dystrophin, Dystrophin-associated proteins, Dystrophin knockout mouse model, Vascular smooth muscle

## Abstract

As humans age, the aorta stiffens, diminishing its essential shock absorber function. This increased stiffness transmits higher pressures to downstream vessels in the kidney, brain, and heart, contributing to hypertension and end-organ damage. Although multiple mechanisms involving extracellular matrix (ECM) remodeling and vascular smooth muscle cell (VSMC) contributions to aortic stiffness have been described, additional molecular players likely remain uncharacterized. Dystrophin (DYS) and the dystrophin-associated proteins (DAPs) are cytoskeletal stabilizers known for protecting skeletal muscle cells from contraction-induced damage but are poorly characterized in VSMCs. Loss of DYS has been linked to myocardial stiffness and carotid artery abnormalities. Here, we investigated the function of DYS in biomechanical properties of the mouse aorta. We demonstrated by immunofluorescence that DYS and DAPs are expressed and colocalize in freshly dissociated murine VSMCs. The mdx model, a known dystrophin knockout mouse model, was used to investigate the consequences of lack of dystrophin expression on aortic geometry and biomechanics. In mdx mice, we observed decreased aortic wall thickness but no significant difference in diameter compared to wild-type (WT) mice. This significant difference in aortic geometry is directly related to ex vivo stress and stiffness. We measured aortic stiffness by high-frequency small-amplitude sinusoidal length perturbation and determined dystrophin is essential to maintain normal stiffness and stress at baseline. This suggests that extracellular matrix components in the mdx aorta are contributing to the increased stress and stiffness at baseline. We also determined that DYS is not required to maintain normal stress and stiffness due to the contractile response to depolarization by high K + or alpha-agonist, phenylephrine-induced contraction. Finally, we revealed by Western blot that DYS and alpha-sarcoglycan, a DAP, expression is decreased in aged vascular smooth muscle. Previous studies show that matrix metalloproteinase-2 (MMP2) can degrade DYS in cardiac tissue and that MMP2 activity increases with age in vascular tissue. While we did not directly assess MMP2 in this study, we propose its role as a hypothesis for future exploration. In summary, this study identifies a novel role for DYS in maintaining aortic mechanical integrity and presents evidence that aging diminishes DYS and DAP protein expression in VSMCs.

**Supplementary Information:**

The online version contains supplementary material available at https://doi.org/10.1007/s11357-025-01947-5.

## Introduction

Dystrophin (DYS) is the largest known human gene and is located on the X chromosome [41]. The DYS protein contains four domains: (1) the actin-binding domain (ABD) at the amino terminal domain, (2) the central rod domain that contains 24 triple helical spectrin-like repeats (SR) combined with 4 hinge domains, (3) the cysteine-rich domain, (4) the carboxy-terminal domain [24]. The DYS protein is known to be associated with the dystroglycan complex (DGC) and thus links the extracellular matrix (ECM) to the underlying actin cytoskeleton [10]. DYS is also known to act to as a cytoskeletal stabilization protein and defends cells against contraction-induced damage [49]. In skeletal muscle, DYS stabilizes the plasma membrane by transmitting forces generated by sarcomeric contraction to the ECM [32, 46, 49]. Skeletal muscle lacking functional dystrophin are mechanically vulnerable; contraction of the cells results in membrane damage [49]. DYS is also an important scaffold for dystrophin-associated proteins (DAPs) including sarcoglycans, syntrophins, dystroglycans, dystrobrevin, neuronal nitric oxide synthase, and caveolins [21, 50]. DAPs provide biomechanical support to the skeletal or cardiac plasma membrane during contraction, and loss of 1 or several of these proteins leads to plasma membrane fragility [50]. DYS has been most studied in the context of the disease muscular dystrophy. More specifically, mutations in the DYS gene lead to either Duchenne Muscular Dystrophy (DMD) or Becker muscular dystrophy [5, 16]. Out-of-frame mutations of 1 or several of the 79 exons in the full-length dystrophin gene results in a lack of a functional protein, causing the DMD phenotype [5, 16].

Vascular smooth muscle (VSM), an important type of smooth muscle, also has been shown to express DYS. Lees et al.. concluded that DYS could be used as a marker of functional cultured rat vascular smooth muscle cells (VSMCs) because it was only expressed in contractile VSMCs and not proliferative VSMCs [25]. Another group, Mancinelli et al. investigated the biomechanical properties of smooth muscle and determined the dystrophic process alters the spontaneous motor activity of the mdx mouse portal vein [28]. There are also marked biomechanical differences of the carotid arteries of two mouse models of muscular dystrophy [12]. This group confirmed that in both models, the dystrophic mice had decreased distensibilities in pressure- diameter tests, elevated axial loads and stresses in axial force-length tests, and decreased in vivo axial stretches compared to wild-type mice [12]. One group conclude that numerous transcripts, including dystrophin, were upregulated with jasplakinolide treatment of VSMC including both known and previously unknown actin-regulated genes [45]. It was also shown that DYS was highly expressed in differentiated smooth muscle compared to synthetic smooth muscle [45]. A mouse model was generated to study smooth muscle and DYS. Ito et al. generated transgenic mdx mice that expresses dystrophin only in smooth muscle (SMTg/mdx) [19]. These SMTg/mdx mice demonstrated that restored DYS expression in the VSM partially corrects the abnormal α-adrenergic vasoconstriction in exercising skeletal muscle [19]. These findings suggest that DYS in VSM might be functionally important. Another group has determined that VSMCs from mdx mice have a dysregulation of intracellular calcium due to overactivation of transient receptor potential canonical channels [27]. This data suggests that the lack of DYS in mdx VSMCs makes these cells more susceptible to contraction-induced damage [27]. Although some groups have studied dystrophin in VSM, the role of this important protein remains to be determined.

Aortic stiffness, a biomechanical property of material and structure, is clinically assessed by pulse wave velocity (PWV). Elevated PWV in the large vessels, such as the aorta, leads to increased transmission of high pressures to small vessels downstream and is proposed to cause remodeling of small vessels and subsequent adverse effects on end-organ function [31]. Increased aortic stiffness is an independent predictor of negative cardiovascular outcomes, including hypertension, kidney disease, stroke, and vascular dementia [43]. Most studies contribute aortic stiffness to both VSMCs and the ECM [3, 4, 15, 37, 47, 51]. There are three mechanisms that have been shown to contribute to VSM stiffness: first, the addition of cross-bridges on contractile filaments in a cyclic manner; second, the remodeling and transmission of stiffness and force through the actin cytoskeleton; and third, the remodeling of focal adhesion proteins coupled to actin cytoskeleton [22, 23, 35]. Although multiple mechanisms of VSMCs contributing to aortic stiffness have been worked out, additional research is still needed. Aortic stiffness is clinically relevant and an independent predictor of negative cardiovascular outcomes; therefore, it is important to understand the mechanisms underlying.

Here, we investigate the expression and functional importance of dystrophin and its associated proteins in the aortic wall and VSMCs, and how their expression is altered by aging. Specifically, we examine the consequences of DYS deficiency using the mdx mouse model, and we assess the expression of DYS and alpha-sarcoglycan in young versus aged wild-type mice. This work addresses a gap in our understanding of cytoskeletal regulation in vascular aging and identifies DYS as a potential molecular mediator of age-associated aortic stiffening.

## Material and methods

### Animals and aortic tissue preparation

C57BL/6J young adult male mice (Jackson Laboratories) 3 months old, C57BL/6J aged adult male mice (National Institute of Aging) 24 months old, and C57BL/10ScSn-*Dmd*^*mdx*^/J (mdx) (Jackson Laboratories) 3 months old were used in this study. All procedures were performed according to a protocol approved by the Boston University IACUC. Animals were sacrificed by overdose of isoflurane in a closed chamber. Aortic tissue was quickly excised, rinse, and placed in an ice-cold oxygenated (95% O_2_ 5% CO_2_) Krebs solution (120 mM NaCl, 5.9 mM KCl, 11.5 mM dextrose, 1.2 mM NaH_2_PO_4_⋅H_2_O, 1.2 mM MgCl_2_⋅6H_2_O, and 2.5 mM CaCl_2_). Axial rings (5 mm length) were cut from the thoracic aorta. Loose perivascular fat was removed from aortic samples. At the end of the protocol, rings were flash frozen in a dry ice, acetone, 10 mmol/L dithithreitol, 10% trichloroacetic acid slurry, and stored at − 80 °C.

### Isolation of single differentiated smooth muscle cells from mouse aorta

Cells were freshly enzymatically dissociated from aortic tissue by a previously published method [22, 29, 39]. Briefly, the aorta was carefully dissected to remove all connective tissue and the endothelium under a dissecting microscope. About 100 mg (wet weight) of aorta tissue was then cut into 2 × 2-mm pieces and placed into a silicon-coated flask containing 7.5 mL Ca^2+^, Mg^2+^-free Hanks’ balanced salt solution (HBSS), ∼100 U/mL type II collagenase (Worthington), ∼ 1.0 U/mL elastase (Roche Diagnostics), and 0.2% BSA (Sigma). The flask was placed in a shaking water bath (∼ 34 °C) under an atmosphere of 100% oxygen for 60 min. The tissue pieces were filtered on a nylon mesh (pore size, ∼ 0.5 mm) and washed with ∼ 12 mL Ca^2+^, Mg^2+^-free HBSS, and 0.2% BSA. The wash buffer containing the dissociated cells was collected and poured over glass coverslips on ice under an atmosphere of 100% oxygen. Cells were allowed to adhere to the coverslips for ∼ 1 h. After the first digestion, additional digestions were performed with 50% less elastase for ∼ 20–30 min.

### Cell staining, immunofluorescence microscopy, and colocalization quantification

dVSMC cells were fixed with 4% paraformaldehyde for 20 min and washed twice with 0.1 mmol/L glycine in 1% BSA Hank’s Balanced Salt Solution (HBSS), permeabilized with 0.1% Triton X-100 for 10 min and blocked with 10% goat serum in 0.05% Triton X-100 and 1% BSA HBSS for 30 min. Cells were incubated overnight with the specific primary antibody in 2% goat serum and 1% BSA HBSS. Cells were washed five times with 0.1 mmol/L glycine in 1% BSA HBSS. Subsequently, cells were incubated with secondary antibody and mounted with fluoroshield with DAPI (Abcam).

For high-resolution deconvolution microscopy, three-dimensional image stacks were acquired with Nikon Eclipse TE 2000-E inverted microscope using a Nikon Plan Apochromat 60X (Numerical Aperture 1.4) oil immersion objective and a charge-coupled device camera (Cool SNAP HQ2, Photometrics). NIS-Element Advanced Research software was used to capture images, and out-of-focus fluorescence was removed by deconvolution of *z*-stacks. The degree of colocalization was quantified from merged images by using Pearson’s correlation coefficient (PCC) [11]. The “colocalization tool” in the Nikon NIS-Element Advanced Research software was used to measure PCC values. For accurate measurement of the PCC, cells were outlined to avoid any unlabeled extracellular regions that can artificially inflate PCC values if included.

### Measurement of aortic geometry and biomechanics

Measurements of aortic geometry as previously described [20, 33, 39]. Axial length, diameter, and wall thickness were recorded for calculation of vessel cross-sectional area (CSA), which was used to measure vessel stress. Axial length and diameter were measured under a light microscope after aortic dissection of unloaded segments in Hanks Buffer. For wall thickness measurements, ~ 1 mm length rings were cut at the proximal and distal end of each aortic ring and incubated in nuclear stain (NucBlue; Life Technologies, Carlsbad, CA, USA) for 30 min. After incubation, individual rings were imaged with fluorescence microscopy for autofluorescence of medial elastin fibers and NucBlue stain via NIS-Elements software (Nikon Instruments, Melville, NY, USA). Approximately 15–20 measurements were recorded for each ring to calculate the average wall thickness. Then, the averages for the proximal and distal rings for each strip were calculated to obtain the wall thickness for the upper and lower thoracic aorta.

Ex vivo aortic stress was measured as previously described [6, 15, 20, 37, 39]. Briefly, thin triangular pieces of wire (0.01-inch diameter) were threaded through the lumen of the aortic ring (4–5 mm) and suspended in an organ bath (50 mL) containing oxygenated (95% O_2_ 5% CO_2_) 50 mL of Krebs solution with 100 μM N(gamma)-nitro-l-arginine methyl ester (l-NAME) at 37 °C. The upper triangle was connected to a computer-controlled motorized lever arm (Dual-Mode Lever Arm System, Model 300 C, Aurora Scientific), which also functions as a force transducer. Aortic rings were stretched to 80% of the initial length (optimal length for force production) and allowed to equilibrate for 30–60 min. After equilibration, the standard Krebs solution was replaced with a depolarizing 51 mM KCl Krebs solution (30 mM NaCl, 51 mM KCl, 11.5 mM dextrose, 1.2 mM NaH2PO_4_.H_2_O, 1.2 mM MgCl_2_.6H_2_O, and 2.5 mM CaCl_2_) to check the viability of the tissues. After 15 min, the KCl Krebs solution was replaced with standard Krebs solution, and the muscle was allowed to relax for 30 min. Depolarizing KCl Krebs solution was repeated along with return to Krebs and relaxation for 30 min.

After relaxation, the muscle was incubated with 10 μM phenylephrine-Krebs solution to assay the smooth muscle cell contraction amplitude. Stress was calculated as force Δ*F* divided by the cross-sectional area (*A* = 2*h*ι where *h* is the wall thickness and ι is the axial length of the ring). Myography data was collected in LabChart (AD Instruments, Dudedin, New Zealand).

Ex vivo aortic stiffness was measured as previously described [6, 15, 20, 37, 39]. Briefly, thin triangular pieces of wire (0.01-inch diameter) were threaded through the lumen of the aortic ring (4–5 mm) and suspended in an organ bath (50 mL) containing oxygenated (95% O_2_ 5% CO_2_) 50 mL of Krebs solution with 100 μM N(gamma)-nitro-l-arginine methyl ester (L-NAME) at 37 °C. The upper triangle was connected to a computer-controlled motorized lever arm (Dual- Mode Lever Arm System, Model 300 C, Aurora Scientific), which also functions as a force transducer. Aortic rings were stretched to 80% of the initial length (optimal length for force production) and allowed to equilibrate for 30–60 min. After equilibration, the standard Krebs solution was replaced with a depolarizing 51 mM KCl Krebs solution (30 mM NaCl, 51 mM KCl, 11.5 mM Dextrose, 1.2 mM NaH2PO_4_.H_2_O, 1.2 mM MgCl_2_.6H_2_O, and 2.5 mM CaCl_2_) to check the viability of the tissues. After 15 min, the KCl Krebs solution was replaced with standard Krebs solution, and the muscle was allowed to relax for 30 min. Depolarizing KCl Krebs solution was repeated along with return to Krebs and relaxation for 30 min.

After relaxation, the muscle was incubated with 10 μM phenylephrine-Krebs solution to assay the smooth muscle cell contraction amplitude. Aortic stiffness was determined by high-frequency (40 Hz), small-amplitude (1%) sinusoidal length perturbations. Aortic tissue stiffness is defined as the change in stress divided by the change in strain in response to the sinusoidal oscillations, (Δ*F*/*A*)/Δ*L*/*L*_0_) where Δ*F* is the amplitude of force output during cyclic stretches, *A* is the cross-sectional area, Δ*L* is the amplitude of cyclic length changes, and *L*_0_ is the optimal length. Myography data was collected in LabChart (AD Instruments, Dudedin, New Zealand).

### RNA quantification, reverse transcription PCR, and qRT-PCR

RNA was isolated as previously published [33]. Briefly, quick-frozen aortic strips were homogenized in Trizol by a beat-beating technology (Precellys 24; Bertin Technologies, Montigny-le-Bretonneux, France). The RNeasy Plus Kit was followed according to manufacturers’ instructions (Qiagen, Hilden, Germany). RNA was quantified by NanoDrop (Agilent Technologies, Santa Clara, CA, USA) and a 260/280 ratio of > 1.8 was required for further study.

RNA was reverse transcribed by the TaqMan Reverse Transcription kits for the quantification of gene expression, according to the manufacturer’s instructions.

For mRNA analysis, TaqMan mouse gene expression assays were used for DYS (Mm01216951_m1 Applied Biosystems by Thermo Fisher Scientific, Waltham, MA) and ΑSG (Mm00486068_m1 Applied Biosystems by Thermo Fisher Scientific, Waltham, MA). Gene expression was normalized to GAPDH (Mm99999915_g1). Samples were combined with PCR grade water (Thermo Fisher Scientific, Waltham, MA) and Taqman Universal PCR Master Mix (Applied Biosystems by Thermo Fisher Scientific, Waltham, MA). Quantitative reverse transcription polymerase chain reaction (qRT-PCR) was performed on the QuantStudio 5 Real-Time PCR system (Applied Biosystems by Thermo Fisher Scientific, Waltham, MA). All fold changes were calculated by the 2^−ΔΔCt^ method as described previously and compared with young mice [26].

### Western blotting

Aortic tissues were homogenized as previously described [34]. Aortic tissues were placed in Precellys cell lysis tubes containing 30 μL of homogenization buffer with protease and phosphatase inhibitor cocktail (Thermo Fischer Scientific Cat # 78440). The tubes were placed in a bead beater and 4 cycles (1 cycle: 2 × 25 s on) and were run at 5000 rpm with an interval of 3 min between each cycle at 4 °C. Tissue homogenates were cleared by centrifugation for 10 min at 13,000 rpm, 4 °C. Protein concentration was estimated using a Bio-Rad DC protein assay kit per the manufacturer’s instructions.

Western blotting was performed to detect proteins using antibodies. Twenty-five micrograms of protein was resolved by SDS-PAGE and transferred to a nitrocellulose membrane (Bio-Rad, Hercules, CA). For high molecular weight proteins, wet electrotransfer was used 4 h at 240 mA at room temperature with ice pack (200 mM glycine, 25 mM Tris, 10% methanol, 0.1% SDS). For other, non-high molecular weight proteins, semi dry electrotransfer was used for 1.5 h at 120 mA at room temperature as previously published [20, 34, 39]. Membranes were dried at room temperature for 30 min followed by blocking in LI-COR blocking buffer in TBS for 1 h at room temperature. Membranes were incubated in primary antibodies at 4 °C overnight. Following primary antibody incubation, blots were washed with TBS-Tween-20 (0.05%) solution 5 times with an interval of 5 min. Blots were incubated with fluorescently labeled secondary antibodies for 1 h at room temperature. Washes with TBS-Tween-20 (0.05%) solution 5 times with an interval of 5 min were repeated. Finally, protein bands were visualized on Sapphire Western Blot Imager system (Azure Biosystems, Dublin, CA, USA) or Odyssey infrared Imaging System (LI-COR, Lincoln, NE, USA) and densitometric analysis was performed with FIJI. Intensity was adjusted for display purposes, but all analysis was performed on the raw data. For analysis of all protein expression, bands of interest were normalized to GAPDH. Bands were further normalized to young aorta.

### Chemicals and antibodies

All chemicals used in this study were of molecular biology grade and were purchased from either Millipore Sigma or Thermo Fisher Scientific. Reagents are included in table (Table 1).

**Table 1:** Reagents and applications

Reagent/antibody	Host species	Application	Vendor	Catalog number
Dystrophin (DYS) antibody	Rabbit	IF, WB	Abclonal	A1411
Alpha-sarcoglycan (α-SG)	Mouse	IF, WB	Abclonal/Santa Cruz	A19754/sc-271321
Caveolin-1 (Cav1) antibody	Mouse	IF	Santa Cruz Biotechnology	sc-53564
Beta-dystroglycan (β-DG)	Mouse	IF	Santa Cruz Biotechnology	sc-33701
GAPDH antibody (loading control)	Mouse	WB	Sigma Millipore	G9545
Phalloidin (Alexa Fluor 488)	—	IF (actin stain)	Invitrogen/Thermo Fisher	—
NucBlue live cell stain	—	Nuclear staining	Invitrogen/Thermo Fisher	R37605

### Statistics

All results are presented as mean ± SEM analysis was carried out using Prism 10 software (GraphPad, La Jolla, California, USA). Two-tailed Student’s *t* tests were used when comparing values between two experimental groups. The differences were considered significant when *p* < 0.05.

## Results

### Localization and colocalization of DYS and DAPs in dVSMC

**Fig. 1:**
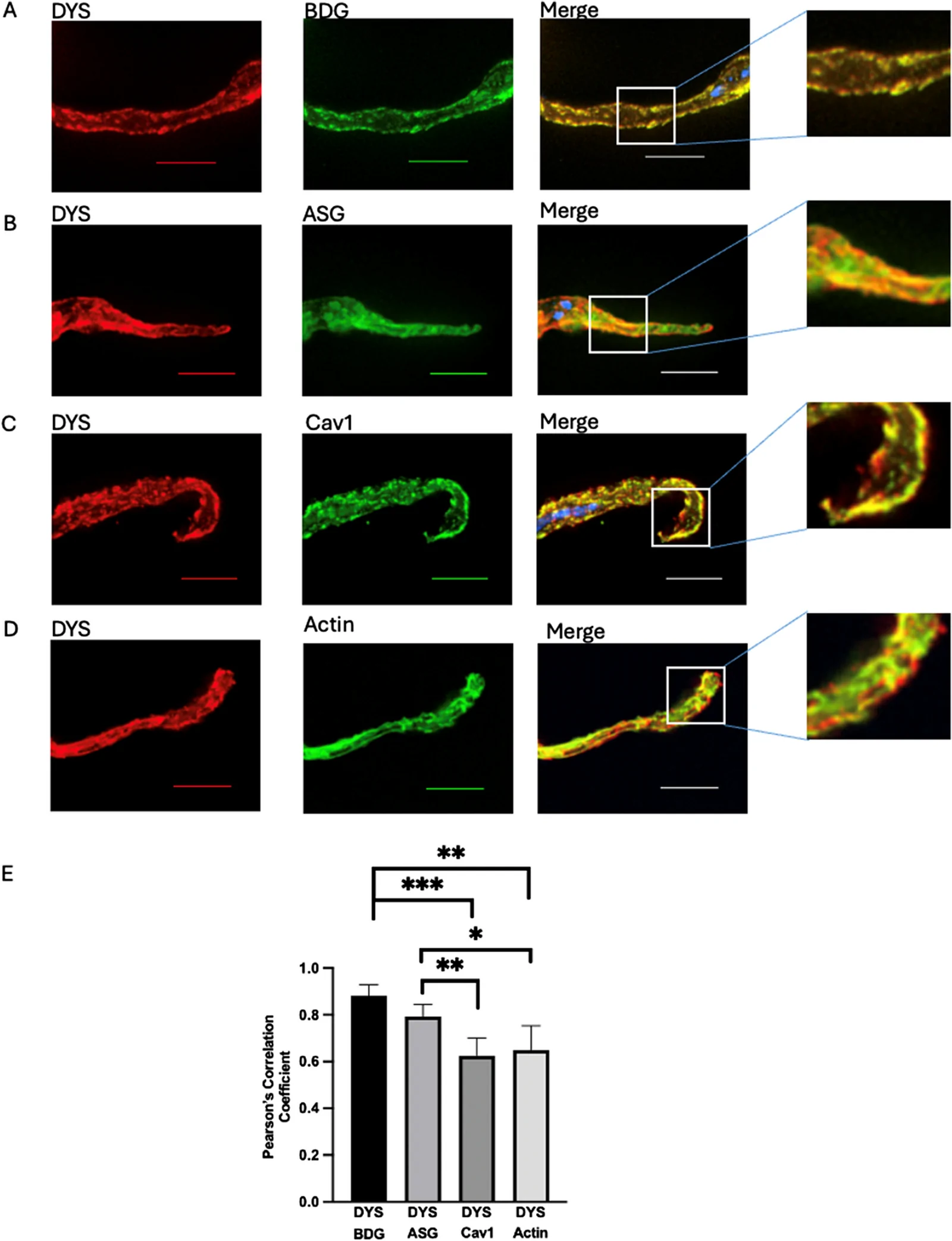
Dystrophin and associated proteins localize to the plasma membrane of murine VSMCs. Subcellular localization of dystrophin, α-sarcoglycan, β-dystroglycan, caveolin 1, and actin. Freshly enzymatically isolated dVSMCs from mouse aortas were fixed and probed with specific antibodies. Deconvolution immunofluorescence microscopy was used to increase the resolution of imagining. **A** Images demonstrate that DYS is localized to the membrane. **B** and **C** BDG and ASG are also localizing to the membrane. Co-labeling was detected with DYS, BDG, and ASG indicated by yellow in merged images. Higher magnification of the merged images can be seen on the right. **D** Partial colocalization and a lack of yellow staining can be observed with DYS and filamentous actin. **E** Summary graph of colocalization using Pearson’s correlation coefficients (PCC). PCC values are expressed as mean ± SEM. Scale bar = 5 μm

DYS is localized to the plasma membrane of all muscles, including cardiac, smooth muscle, and some neurons [1, 2, 9, 17]. DYS is known to be associated with the dystroglycan complex (DGC), a transmembrane complex, and thus links the extracellular matrix (ECM) to the underlying actin cytoskeleton [10]. To study whether DYS localizes to the plasma membrane in VSMCs, we enzymatically dissociated single dVSMCs from the mouse aorta and imaged the cells with high-resolution deconvolution microscopy as previously described [22]. DYS localizes to the plasma membrane as it does in other muscle types (Fig. 1A, B, C, D).

In skeletal muscle, α- and β-dystroglycans are key proteins in the DGC, linking laminin-2 and dystrophin [13]. Sarcoglycans are a family of transmembrane proteins also shown to interact with DYS in skeletal muscle [13]. Caveolins have been hypothesized to interact with dystroglycan and mutations in Cav-3 are associated with types of muscular dystrophy [14]. Sharma et al. demonstrated that Cav-1 interacts with β-dystroglycan in airway smooth muscle [38]. To investigate the localization and colocalization of DYS and these DAPs in mouse aortic cells, we used specific antibodies and found a similar pattern to other muscle types. We then co-stained for the DAPs to allow comparison of colocalization in the dVSMCs. BDG, a transmembrane component of the DGC, is known to bind DYS. We found that DYS colocalizes with BDG at the plasma membrane (Fig. 1A). Similarly, we co-stained for DYS and ΑSG. The merged image shows DYS also colocalizes with ΑSG at the plasma membrane as it does in other muscle types (Fig. 1B).

Next, we investigated the localization of DYS and Cav1. We conclude that DYS and Cav1 partially colocalize in dVSMCs. This is shown in the merged image with partial yellow signal partial red and green signal (Fig. 1C). This indicates that DYS may be interacting with Cav1 through the DGC as it does in airway smooth muscle.

Finally, DYS contains an acting binding domain at the N-terminal end. To investigate whether DYS colocalizing with actin we stained with phalloidin which filamentous actin. We show that DYS and actin partially colocalize in dVSMCs. This is demonstrated in the merged image with partial yellow signal partial red and green signal (Fig. 1D).

To quantitatively determine the degree of colocalization between DYS and DAPs, Pearson’s correlation coefficients (PCC) were calculated using the “Colocalization” tool in Nikon NIS- Element Advanced Research software. PCC values near zero indicate a lack of correlation between fluorescence intensities of two images, whereas a PCC value of 1 indicates a perfect linear relationship between the two images. A high degree of colocalization was indicated in merged images of DYS/BDG and DYS/ΑSG as the PCC values were 0.881 ± 0.047 and 0.793 ± 0.052 respectively. Additionally, significantly less colocalization was indicated in the merged images of DYS/Cav1 and DYS/actin as the PCC values were 0.625 ± 0.076 and 0.649 ± 0.10 respectively (Fig. 1E) (*p* = 0.018, *p* = 0.0242). A total of 20 cells (five from each group) were analyzed for quantification.

### Aortic geometry

**Fig. 2:**
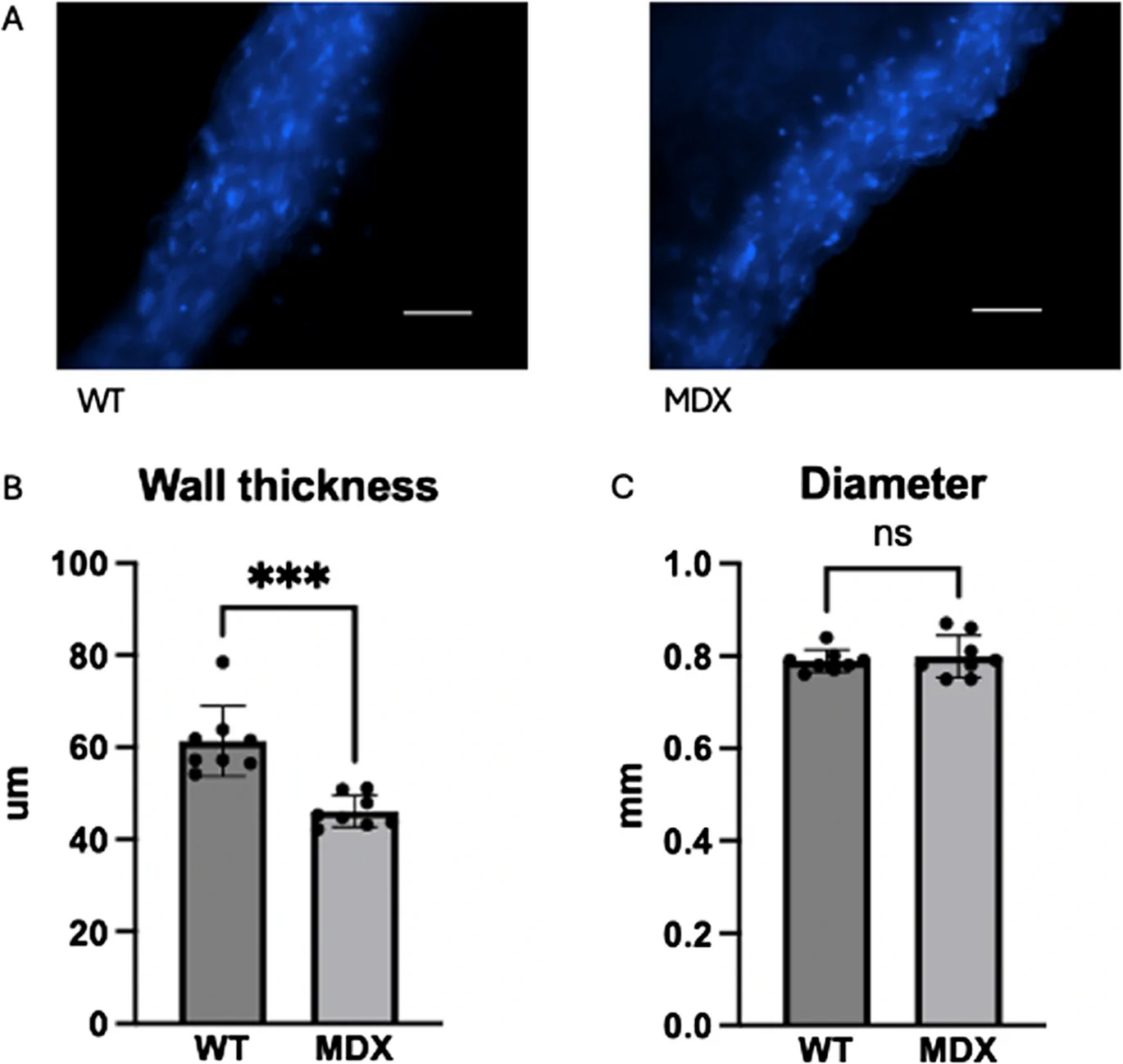
Dystrophin deficiency in mdx mice results in significantly thinner aortic walls without affecting lumen diameter. Aortic geometry of the mdx mouse. **A** NucBlue staining demonstrating wall thickness of aortic rings. Scale bar = 50 μm. **B** Bar graph representing mean ± SEM of wall thickness of young mdx and young WT aorta. The mdx aorta is significantly thinner (*p* = 0.0001). **C** Bar graph representing mean ± SEM of aortic diameter of young mdx and young WT aorta (2C). No significant difference was observed in the diameter of young mdx and young WT aortas (*n* = 8)

Dye et al. studied the biomechanical properties of the carotid arteries in the mdx model as well as another mouse model of muscular dystrophy [12]. This group determined that, in both models, the dystrophic mice had decreased distensibilities in pressure-diameter tests, elevated axial loads and stresses in axial force-length tests, and decreased in vivo axial stretches compared to wild-type mice [12]. Interestingly, they also concluded the percentage of the aortic wall occupied by the smooth muscle appeared to be less in both the *mdx* and the *sgcd −/− *mice compared with wild-type controls, therefore proposing more adventitia and adventitial collagen in the 2 mouse models of muscular dystrophy [11]. To investigate the role of DYS in the aorta the mdx knockout model was used. We compared aortic ring segments from the thoracic aorta of both C57BL/6 J (WT) young adult male mice and C57BL/10ScSn-*Dmd*^*mdx*^/J (mdx) young adult male mice. We show the wall thickness of the mdx aorta (46.15 ± 3.43 μm) is significantly thinner than the WT aorta (61.38 ± 7.64 μm) (*p* = 0.0001, Fig. 2A, B). These results corroborate Dye et al.’s results from the carotid artery [12]. The diameter of the thoracic aorta of the mdx mouse (0.79 ± 0.05) was not significantly different than that of the WT (0.79 ± 0.02) (Fig. 2C). Aortic geometry is demonstrative of the health of the vessel, the functional ability of the vessel as well as greatly influential on biomechanics.

### Ex vivo stress and stiffness

**Fig. 3:**
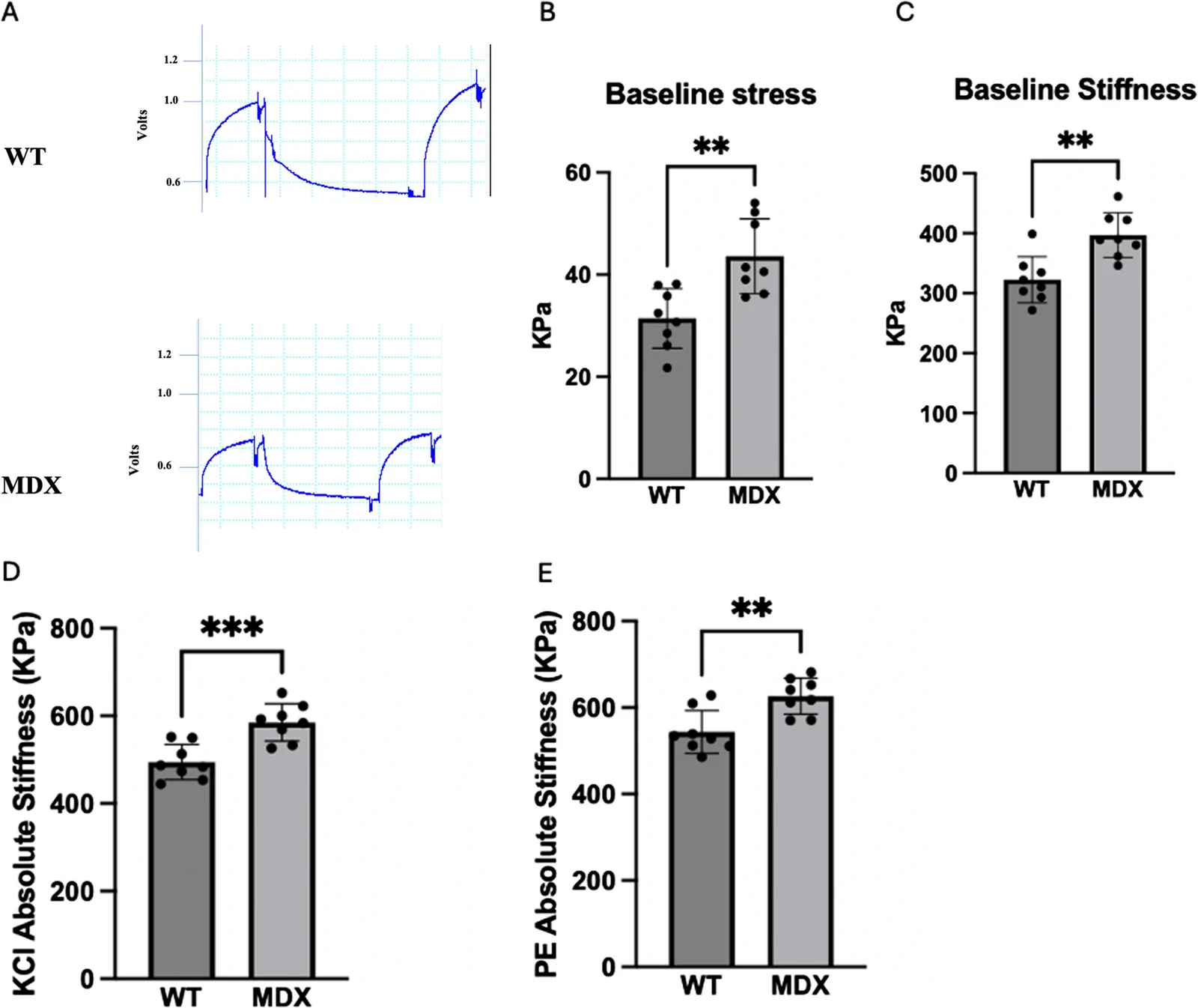
Aortas from mdx mice display significantly increased baseline and contractile-induced stiffness and stress. **A** Myograph tracings of young mdx and young WT aortas connected to controlled motorized lever arm force transducer. Our group previously reported and define baseline force as that experienced by an aortic tissue sample stretched to 80% strain and allowed to equilibrate [15]. **B** Baseline stress was calculated as force Δ*F* divided by the cross-sectional area (*A* = 2*h*ι where *h* is the wall thickness and ι is the axial length of the ring). Bar graph representing mean ± SEM of baseline stress demonstrates the mdx aorta has significantly elevated baseline stress (*p* = 0.0025). **C** Aortic tissue stiffness is defined as the change in stress divided by the change in strain in response to the sinusoidal oscillations, (Δ*F*/*A*)/Δ*L*/*L*_0_) where Δ*F* is the amplitude of force output during cyclic stretches, *A* is the cross-sectional area, Δ*L* is the amplitude of cyclic length changes, and *L*_0_ is the optimal length. Bar graph representing mean ± SEM of baseline stiffness demonstrating significantly increased baseline stiffness in the young mdx aorta compared to young WT (*p* = 0.0015). **D** Absolute stiffness is defined as the stiffness without normalizing for the baseline. Bar graph representing mean ± SEM of KCl absolute stiffness demonstrates the mdx aorta has significantly elevated absolute stiffness (*p* = 0.0007). **E** Bar graph representing mean ± SEM of phenylephrine-induced absolute stiffness demonstrates the young mdx aorta has significantly elevated absolute stiffness (*p* = 0.0027) (*n* = 8)

To investigate biomechanical differences of the mdx aorta, we performed ex vivo stress and stiffness [6, 15, 20, 37, 39]. Briefly, baseline stress was calculated by normalizing force to cross-sectional area of unloaded tissue (stress = force/area (area = 2 × wall thickness × axial length). Our group previously reported and define baseline force as that experienced by an aortic tissue sample stretched to 80% strain and allowed to equilibrate [15]. We show baseline stress is significantly increased in the mdx aorta (43.61 ± 7.32 kPa) compared to WT (31.44 ± 5.85 kPa) (*p* = 0.0025) (Fig. 3B).

The baseline stiffness (elastic modulus) was measured as the resultant ratio of change in stress to change in strain in response to HFLA (1%) changes in length, as a sinusoidal oscillatory stretch superimposed upon optimal length. Changes in strain were kept small to cause negligible distress of the tissue. We show baseline stiffness is significantly increased in the mdx aorta (397.1 ± 37.4 kPa) compared to WT (322.5 ± 38.6 kPa) (*p* = 0.0015) (Fig. 3C). These significant differences of baseline stress and stiffness suggest that the ECM in the mdx aorta is contributing to the biomechanical differences. This agrees with previously published literature that determines in skeletal muscle the linking of the actin cytoskeletal to the ECM to be the essential role of DYS [10]. In the mdx aorta, the intracellular to ECM link is broken and thus results in biomechanical differences observed.

We also investigated ex vivo stress and stiffness in the mdx aorta due to VSMC contraction. We determined there were no difference in increased stiffness or stress due to contractile response to depolarization by high K + or alpha-agonist phenylephrine-induced contraction, between mdx and WT (supplemental Figs. 1 and 2). This suggests that the lack of dystrophin does not affect the mechanisms underlying VSM contraction. However, there were significant differences of absolute stiffness of contractile response to depolarization by high K + and phenylephrine-induced contraction. Absolute stiffness is defined as the stiffness without normalizing for the baseline. We show significantly increased absolute stiffness due to contractile response to depolarization by high K + in the mdx aorta (584.9 ± 42.5 kPa) compared to the WT aorta (494.6 ± 40.4 kPa) (*p* = 0.0007) (Fig. 3D). Similarly, phenylephrine-induced absolute stiffness was significantly increased in the mdx aorta (626.6 ± 41. 5 kPa) compared to the WT aorta (543.5 ± 49.7 kPa) (*p* = 0.0027) (Fig. 3E). These findings confirm previously shown data that the baseline stiffness of the mdx aorta is increased (Fig. 3A, B, C).

### DYS mRNA and protein expression with aging

**Fig. 4:**
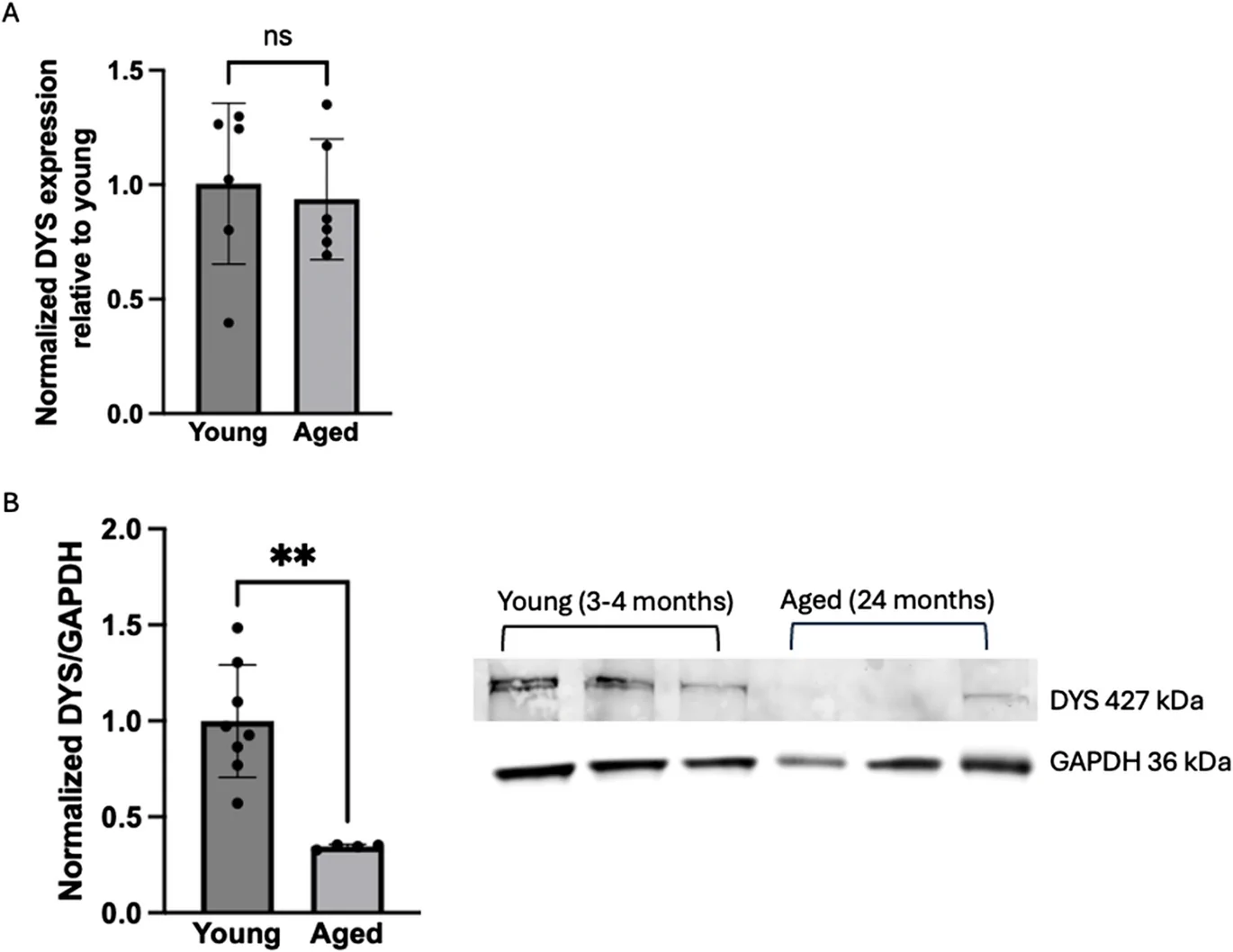
Dystrophin protein expression, but not mRNA, decreases in aged VSMCs, suggesting post-transcriptional regulation. Dystrophin mRNA and protein expression in WT VSM young and aged. **A** Bar graph representing mean ± SEM QRT-PCR for dystrophin mRNA demonstrates no significant difference between aged VSM and young VSM. **B** Western blot demonstrates significantly decreased dystrophin protein expression in aged VSM compared to young (*p* = 0.0014) (*n* = 6)

DYS protein expression has been shown to be decreased with age in both skeletal muscle and cardiac muscle [18, 42]. We show by qRT-PCR DYS mRNA does not change with age (Fig. 4A). This trend is the same that Hughes et al. demonstrated in the skeletal muscle [18]. This group explains this possible discrepancy with dystromirs, muscle-specific miRNAs [18]. Hughes et al. demonstrated a significant increase in dystromir expression with age in skeletal muscle [18]. We therefore hypothesize that post-transcriptional regulation is accounting for the lack of consistency between mRNA and protein results.

We demonstrate by Western blot that DYS protein expression is significantly decreased with age in VSM (*p* = 0.0014) (Fig. 4B). Our findings agree with previously published papers demonstrating DYS decreases in other muscle types with age [18, 36, 42]. These data suggest that the link of the actin cytosletelon to the ECM through dystrophin may be lost or reduced with aging in VSM.

### DAPs mRNA and protein expression with aging

**Fig. 5:**
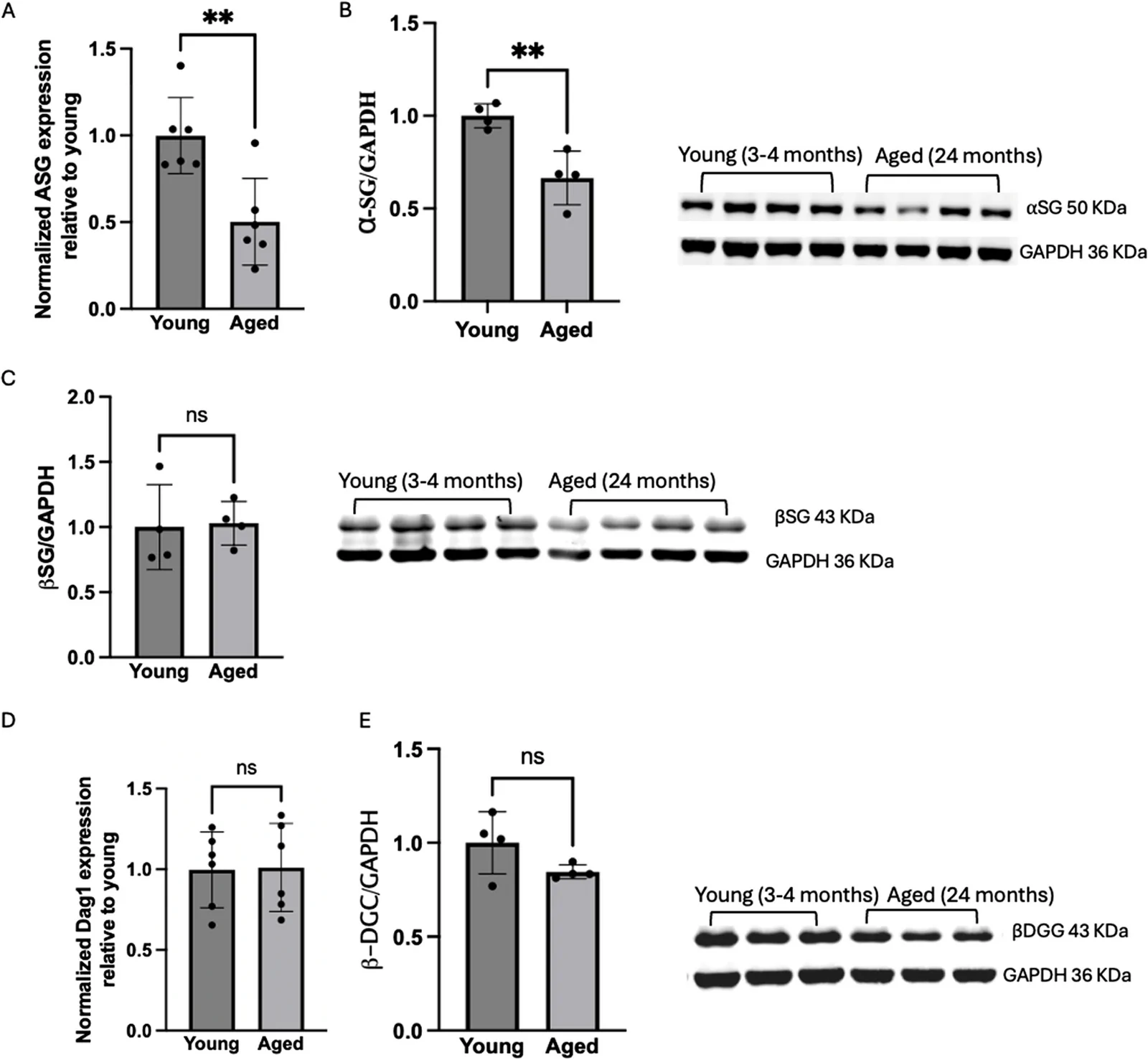
Aging selectively reduces alpha-sarcoglycan expression in VSMCs, while other DAPs remain unchanged. **A** QRT-PCR demonstrates significantly decreased α-sarcoglycan mRNA expression in aged WT VSM compared to young (*p* = 0.0044). **B** Western blot confirms significantly decreased ASG protein expression in aged WT VSM compared to young (*p* = 0.0054). **C** Western blot reveals β-sarcoglycan protein expression remains unchanged with age in VSM. **D** QRT-PCR demonstrates dystroglycan mRNA expression remains unchanged with age in VSM. **E** Western blot reveals beta-dystroglycan protein expression remains unchanged with age in VSM (*n* = 6)

There is very little research on DAPs expression with aging. One group concluded that β-dystroglycan, α-sarcoglycan, and α-dystroglycan expression was altered in aged rat skeletal muscle when compared to young [36]. Our finding that DYS protein expression was affect by aging in VSM led us to investigate the DAPs. We show by QRT-PCR ΑSG mRNA expression significantly decreases with aging in VSM (*p* = 0.0044) (Fig. 5A). We also demonstrate by western blot that ΑSG protein expression is significantly decreased in aged VSM compared to young (*p* = 0.0054) (Fig. 5B). Our group has also previously shown that Cav1 expression, which may be acting as a DAP in VSM, is also decreased in aged VSM [33]. Taken together, these findings establish that in the aged VSM not only is DYS protein expression decreased but some of the DAP expression decreases as well.

There is assumed to be redundancy in the DAPs; particularly the sarcoglycans. The sarcoglycan family is comprised of five transmembrane proteins: *α* (50 kDa, also called adhalin), *β* (43 kDa), *γ* (35 kDa), *δ* (35 kDa), and *ϵ* (50 kDa) [13]. Some sarcoglycans have been shown to be expressed in smooth muscle while other have been shown to be strictly expressed in skeletal muscle [40, 52]. Our findings of ΑSG expression led us to investigate other DAPs. We show that βSG protein expression is not affected in the aged VSM compared to young (Fig. 5C). This finding may suggest that other sarcoglycans can compensate for the decrease in ΑSG with age. Further study is needed to investigate the other sarcoglycans in VSM.

The widely expressed α- and β-dystroglycans are key proteins in the DGC, linking laminin-2 and dystrophin [13]. Both dystroglycans are from a single post-translationally modified polypeptide and are glycosylated prior to being sorted to their extracellular and transmembrane locations [13]. We show by QRT-PCR DAG1 mRNA expression is not altered in the aged VSM compared to young VSM (Fig. 5D). We also show by Western blot the same trend; βDG protein expression is not altered in the aged VSM compared to young VSM (Fig. 5E). In summation, the dystroglycan complex proteins are not affected by aging in the VSM.

## Discussion

**Fig. 6:**
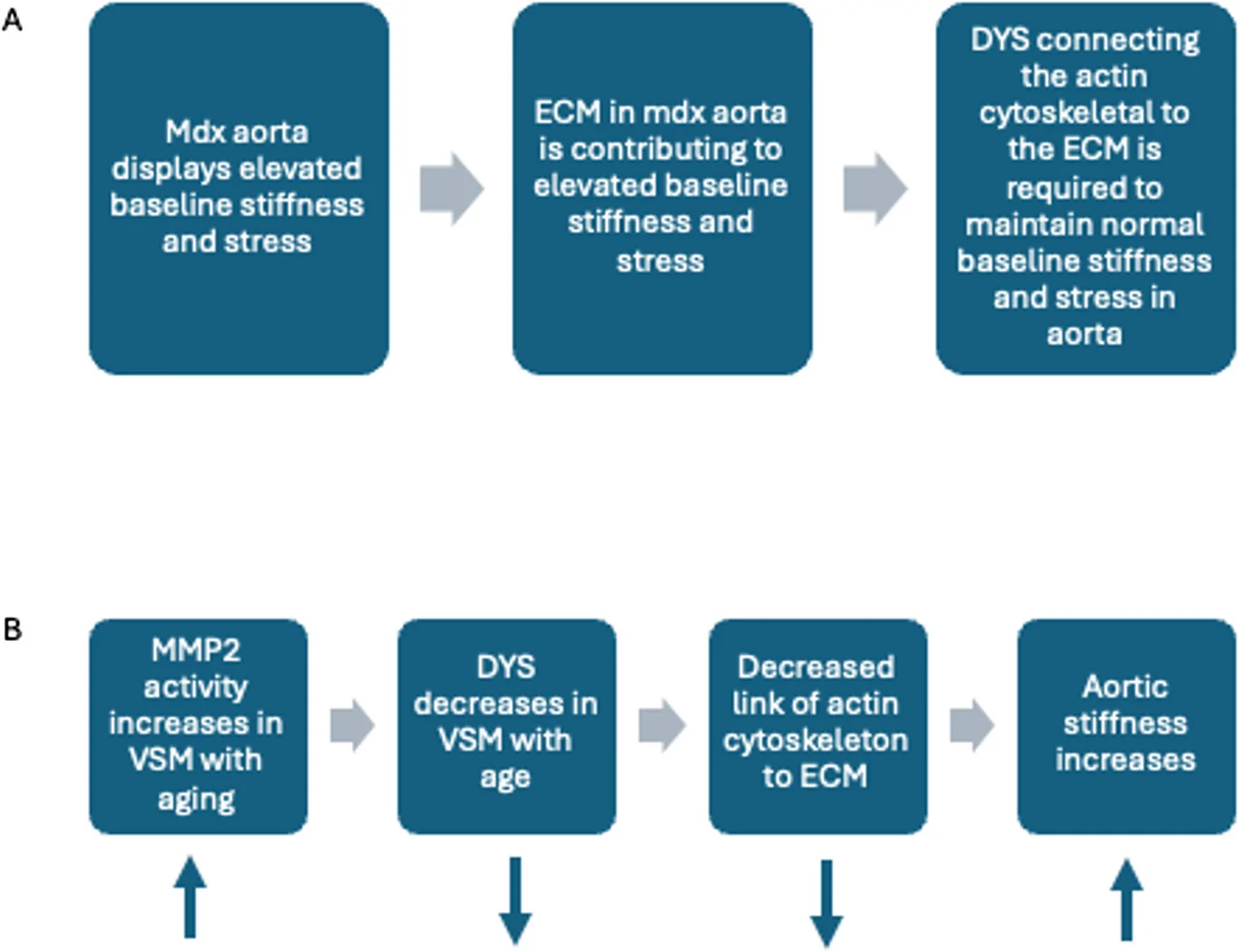
Proposed model: loss of dystrophin with age weakens VSM cytoskeletal-ECM linkage, contributing to aortic stiffness. **A** The mdx aorta displays increased baseline ex vivo stiffness and stress. This suggests the link DYS provides from the actin cytoskeleton to lamin in the ECM is required to maintain normal baseline stiffness and stress in the aorta. **B** Other groups have shown that MMP2 degrades DYS in cardiac tissue [7]. Additionally, other groups have demonstrated that MMP2 activity increases with age in the VSM [30, 48]. It is not yet confirmed whether MMP may be degrading DYS in VSM but taken together with our data demonstrating DYS protein expression is significantly decreased in aged VSM, we hypothesize the mechanism of degradation may be the same of that shown in cardiac muscle, i.e., MMP2. Thus collectively, we hypothesize that increased MMP2 activity results in decreased DYS protein expression in the VSM, therefore degrading the link of the actin cytoskeleton to the ECM and contributing to aortic stiffness with age

Both the *mdx* mouse model and patients with Duchenne muscular dystrophy (DMD) are known to have elevated resting heart rates [16], Thomas, Morgan et al. 2012). Elevated resting heart rate has been associated with increased arterial stiffness (Logan and Kim 2016). In addition, loss of dystrophin has been linked to increased myocardial stiffness (Donato, Buchholz et al. 2017). Taken together, these findings may explain how dystrophin (DYS) contributes to aortic stiffness. In this study, we demonstrate significant differences in aortic geometry and biomechanics between *mdx* and wild-type (WT) aortas. Our results are consistent with previous findings in vascular smooth muscle (VSM) by Dye et al. in the carotid arteries and Mancinelli et al. in the portal vein [12, 28]. A key finding of this study is that *mdx* aortas exhibit increased baseline stiffness and stress compared to WT. This suggests that extracellular matrix (ECM) composition, rather than VSMC contraction-induced stiffness, plays a larger role in the observed differences. Therefore, the link dystrophin provides between the actin cytoskeleton and laminin in the ECM is essential for maintaining normal baseline stiffness and stress in the aorta (Fig. 6A).

Our study is one of the few that have examined the role of dystrophin in smooth muscle. Lees et al. demonstrated that DYS can serve as a marker of functional cultured rat VSMCs [25]. They also reported increased DYS expression in the rat aortic arch, along with greater contractility compared to the aortic diaphragm (Lees, Fabbrizio et al. 1995). This difference in contractility may be attributed in part to DYS, but also to aortic geometry, as the arch is thicker than the diaphragm. Other studies support our findings. Sharma et al. observed significant reductions in contractile protein markers in dystrophin-deficient airway smooth muscle cells (Sharma, Basu et al. 2014). They also found that tracheal rings from *mdx* mice showed significantly reduced isometric contraction in response to methacholine (Sharma, Basu et al. 2014). Similarly, Turczynska et al. reported a 30% decrease in contractile response to high potassium (K⁺) depolarization in *mdx* carotid arteries compared to controls [44]. Although we did not observe a difference in contractile stress or stiffness in response to high K⁺ in the *mdx* aorta, this discrepancy may be due to vessel-specific factors such as geometry or baseline stiffness. Turczynska et al. also found no difference in the F/G actin ratio between *mdx* and WT aortas, concluding, as we do, that the loss of dystrophin compromises the physical anchoring of the cytoskeleton to the plasma membrane, impairing force generation in VSMCs [44].

While the mechanism of dystrophin degradation is not fully understood, Buchholz et al. showed that matrix metalloproteinase 2 (MMP2) can degrade dystrophin in cardiac tissue [7]. MMP activity increases with aging and has been documented in VSM [30, 48]. Although it is not yet confirmed whether MMP2 degrades dystrophin in VSM, our data show that DYS protein expression significantly decreases in aged VSM, suggesting a similar degradation mechanism may be at play. We hypothesize that increased MMP2 activity leads to decreased DYS expression in VSM, thereby weakening the cytoskeleton-ECM linkage and contributing to age-related aortic stiffness (Fig. 6B).

One significant limitation of this study is the absence of direct measurements of arterial blood pressure. While we observed clear differences in aortic geometry and biomechanical properties between *mdx* and WT mice, without corresponding blood pressure data, it is difficult to fully assess the physiological consequences of increased aortic stiffness. Blood pressure measurements would have provided critical insight into the hemodynamic relevance of our findings and are necessary to support our central hypothesis regarding the impact of dystrophin deficiency on vascular function. Future studies incorporating in vivo assessments of blood pressure will be essential to better understand the interplay between vascular stiffness and systemic cardiovascular outcomes in both aging and dystrophic models.

Dystrophin is most extensively studied in the context of muscular dystrophy. Mutations in the DYS gene lead to either Duchenne muscular dystrophy (DMD) or Becker muscular dystrophy [5, 16]. Out-of-frame mutations in one or more of the 79 exons of the dystrophin gene result in a nonfunctional protein and the characteristic dystrophic phenotype. Dystrophin-associated proteins (DAPs) are also implicated; loss of any sarcoglycan subunit disrupts the entire complex, resulting in various forms of limb-girdle muscular dystrophy [8]. Duchenne muscular dystrophy remains fatal and currently incurable [21].

Another significant limitation of this study is our exclusive use of male mice. The dystrophin gene (DMD) is located on the X chromosome, meaning that sex-based biological differences could influence both dystrophin expression and the progression of age-related vascular changes. While Duchenne muscular dystrophy is a sex-linked disorder primarily affecting males, the role of dystrophin in female vasculature—especially concerning potential mosaicism in X chromosome inactivation—warrants further investigation. Therefore, our findings may not be fully generalizable to female populations, highlighting the need for future studies to include both sexes to comprehensively evaluate the role of dystrophin in vascular aging.

A third limitation of this study is that it does not assess the changes associated with aging in the *mdx* model itself. While the *mdx* mouse is an invaluable tool for studying the functional consequences of dystrophin deficiency, it is not an accurate representation of the normal aging process observed in the human population with Duchenne muscular dystrophy. Therefore, we used the young *mdx* mouse primarily as a model of chronic dystrophin deficiency to understand the protein’s direct role in biomechanical stability, rather than as a model for age-related pathology.

Current DMD therapies include gene transfer and exon-skipping strategies (Patterson, Conner et al. 2023). Interestingly, ACE inhibitors and angiotensin receptor blockers (ARBs) are also first-line treatments in early disease management, addressing cardiovascular symptoms and possibly the increased aortic stiffness seen in *mdx* mice. Our findings carry dual significance: first, the vascular effects of muscular dystrophy warrant greater attention in therapeutic planning; and second, our results align with those of Ito et al., who generated a transgenic mouse expressing dystrophin only in smooth muscle and showed that VSM-specific restoration of DYS partially corrected abnormal α-adrenergic vasoconstriction in exercising skeletal muscle [19].

Finally, our data suggest that dystrophin is also important in aging VSM. Restoring DYS expression in aged vessels may improve aortic stiffness—an important predictor of adverse cardiovascular outcomes.

## Supplementary Information

Below is the link to the electronic supplementary material.

Supplementary file1 (PDF 125 KB)

## Data Availability

All data supporting the findings of this study are available within the article and its supplementary materials.
