# Supplementary material for: Dystrophin and dystrophin association protein expression decreases with age in vascular smooth muscle
Source: GeroScience. 2025 Nov 1;48(4):5597–612. doi: 10.1007/s11357-025-01947-5 (PMC13575029; doi:10.1007/s11357-025-01947-5)
Supplement: Supplementary file 1 [file 11357_2025_1947_MOESM1_ESM.pdf]

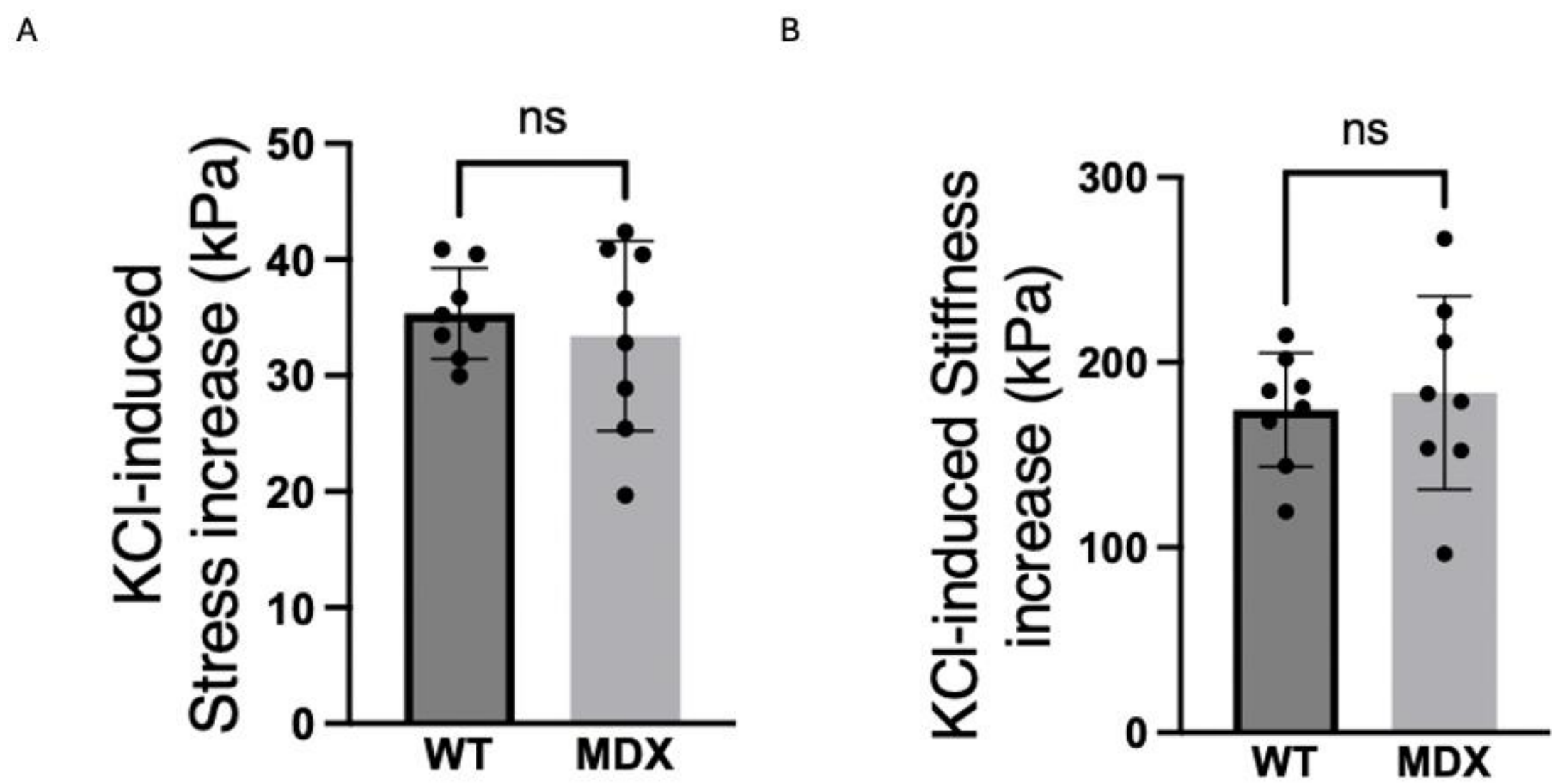

Supplemental Figure 1: The mdx aorta displays no differences in potassium chloride induced stress and stiffness

A) KCl-induced stress was calculated as force ΔF divided by the cross-sectional area ($A = 2h\iota$ where h is the wall thickness and ι is the axial length of the ring). Bar graph representing mean ± SEM of baseline KCl-induced demonstrates no difference between the *mdx* aorta and WT aorta **B)** Aortic tissue stiffness is defined as the change in stress divided by the change in strain in response to the sinusoidal oscillations, $(\Delta F/A)/\Delta L/L_0)$ where ΔF is the amplitude of force output during cyclic stretches, A is the cross-sectional area, ΔL is the amplitude of cyclic length changes, and L_0 is the optimal length. Bar graph representing mean ± SEM of KCl-induced stiffness demonstrates no difference between the *mdx* aorta and WT aorta. (n=8). SEM = standard error of mean.

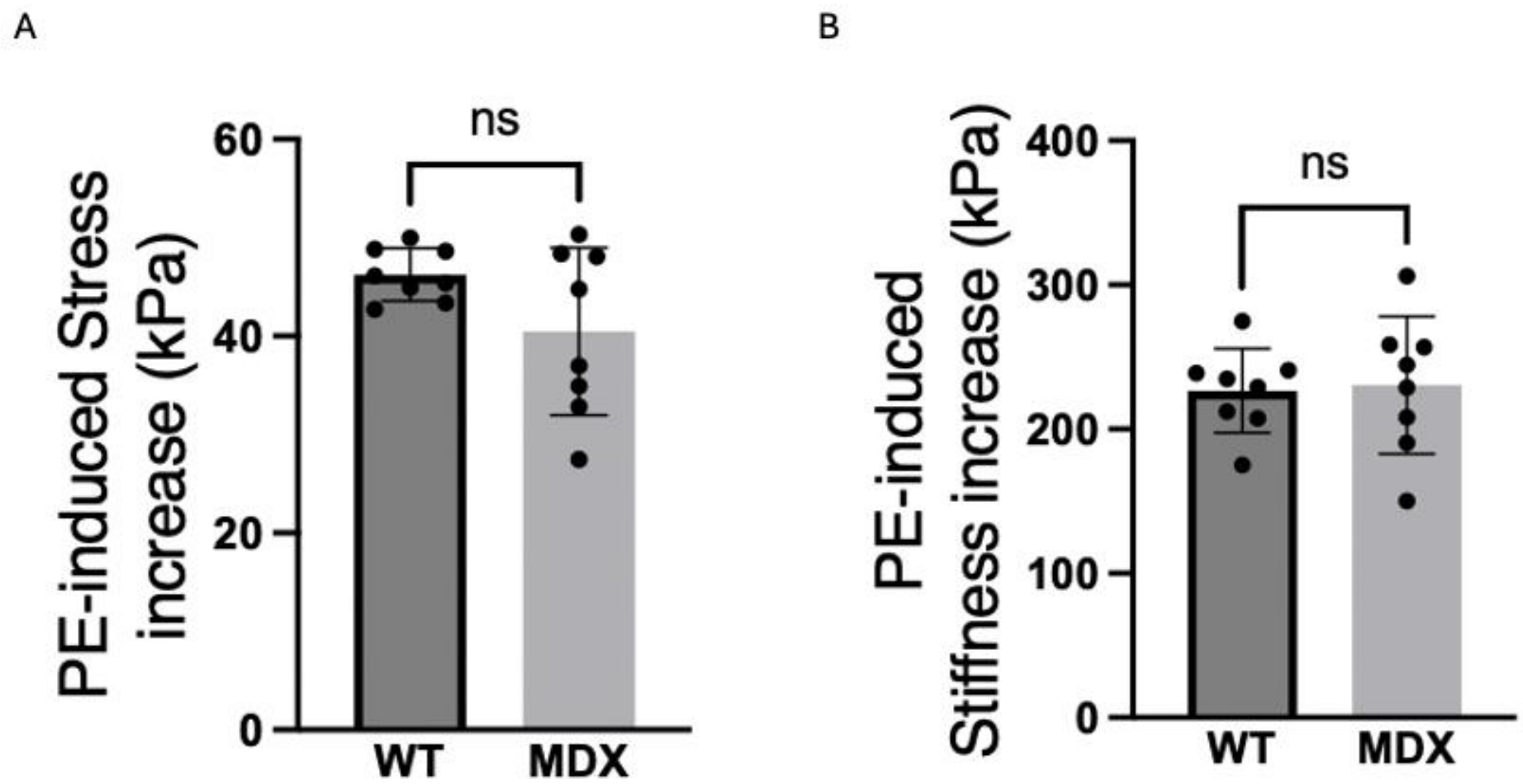

Supplemental Figure 2: The mdx aorta displays no differences in phenylephrine induced stress and stiffness

A) PE-induced stress was calculated as force Δ*F* divided by the cross-sectional area (*A* = 2*h*ι where *h* is the wall thickness and ι is the axial length of the ring). Bar graph representing mean ± SEM of baseline PE-induced demonstrates no difference between the *mdx* aorta and WT aorta **B)** Aortic tissue stiffness is defined as the change in stress divided by the change in strain in response to the sinusoidal oscillations, $(\Delta F/A)/\Delta L/L_0$) where Δ*F* is the amplitude of force output during cyclic stretches, *A* is the cross-sectional area, Δ*L* is the amplitude of cyclic length changes, and L_0 is the optimal length. Bar graph representing mean ± SEM of PE-induced stiffness demonstrates no difference between the *mdx* aorta and WT aorta. (n=8). SEM = standard error of mean.
